# The Genome Sequence of Brucella intermedia DF13, a 2,4-Dichlorophenoxyacetic Acid-Degrading Soil Bacterium Isolated in Brazil

**DOI:** 10.1128/mra.01105-21

**Published:** 2022-03-28

**Authors:** Sheila da Silva, Barbara Alvarenga Peckle, José Roberto de Assis Ribeiro, Selma Soares de Oliveira, Kayo Bianco, Maysa Mandetta Clementino, Ida Carolina Neves Direito, Andrew Macrae

**Affiliations:** a Programa pós-graduação de Biotecnologia Vegetal e Bioprocessos da Universidade Federal do Rio de Janeiro, Rio de Janeiro, RJ, Brazil; b Instituto de Microbiologia Paulo de Góes da Universidade Federal do Rio de Janeiro, Rio de Janeiro, RJ, Brazil; c Laboratório de Micro-organismos de Referência, Instituto Nacional de Controle de Qualidade em Saúde, Fundação Oswaldo Cruz, Manguinhos, Rio de Janeiro, RJ, Brazil; d Laboratório de Biotecnologia Ambiental (LBA) do Centro Universitário Estadual da Zona Oeste (UEZO), Rio de Janeiro, RJ, Brazil; Loyola University Chicago

**Keywords:** *Ochrobactrum intermedium*, *Brucella intermedia*, bioremediation, biodegradation, 2,4-dichlorophenoxyacetic acid (2,4-D), soil

## Abstract

Brucella intermedia/Ochrobactrum intermedium strain DF13 was isolated from Brazilian soil and is able to degrade 2,4-dichlorophenoxyacetic acid (2,4-D). Here, we report on its genome sequence, with 4,570,268 bp and a 57.8% G+C content.

## ANNOUNCEMENT

Members of the genus Brucella are Gram-negative aerobic bacilli (1), belonging to the *Brucellaceae* family (2, 3), and are known as human and animal pathogens (2). Based on recent and thorough genome comparisons, the genus *Ochrobactrum* has been reclassified and its species included within the genus Brucella (4, 5). Consequently, strain DF13, originally classified as Ochrobactrum intermedium, is synonymous with Brucella intermedia. Ochrobactrum intermedium is known to biodegrade organic pollutants (6) as well as organophosphorus pesticides (7, 8). The genus *Ochrobactrum* was described as capable of degrading the pesticide 2,4-dichlorophenoxyacetic acid (2,4-D) (9). Based on our interests in biodegradation, we report on this strain’s genome. The strain was isolated from topsoil, at a depth of 0 to 20 cm, from Fazendinha Agroecológica-SIPA, located at 43°40′00″W and 22°44′30″S. For isolation, a dilution (10^−1^) of 10 g of the soil was performed with 0.1% NaCl (wt/vol), 0.1% (wt/vol) sodium pyrophosphate, and 0.1% (vol/vol) Tween 20 after incubation at 30°C and centrifuged at 180 rpm for 20 min. Then, serial dilution (10^−2^ to 10^−7^) in 0.85% saline was performed. A total of 100 μL of each dilution was plated on a 2,4-D, MEMB selective medium (10). The strain was maintained and grew in a minimal mineral medium with 2,4-D as the sole carbon source. A single colony grown on LB medium at 28°C was used for DNA extraction using the PureLink genomic DNA minikit (Invitrogen) and was quantified with a Qubit fluorometer.

The genomic paired-end library (2 × 300 bp) with 550-bp medium-sized inserts was built using the Nextera XT DNA library preparation kit (Illumina, San Diego, CA) and sequenced on Illumina MiSeq (INCQS; Fiocruz, Manguinhos, RJ, Brazil) using the MiSeq reagent kit V3 (600 cycle). FastQC v0.11.9 (11) and Trim Galore v0.6.6 (12) tools were used to check and trim at a cutoff of >30 for quality Phred score. Genome assembly was performed combining *de novo* and mapping assembly techniques. SPAdes v3.15.0 (13) was used for the *de novo* assembly and the output was used for assembly with MeDuSa v1.6 (14) using 27 Brucella intermedia reference genomes available from the NCBI. CheckM v1.4.0 (15) and QUAST v.5.0.2 (16) were used to check assembly quality and contamination. Genomic annotation was made with NCBI Prokaryotic Genome Annotation Pipeline (PGAP) v5.3 (17–19). Taxonomic identification was based on the Average Nucleotide Identity (ANI) v3.8.3, Pairwise Tetra-correlation (TETRA) v3.8.3, and Tetra Correlation Search (TCS) v3.8.3 from JSpeciesWS (20). The sequencing resulted in 1,036,905 raw reads (602,099,110 bp). SPAdes resulted in 53 contigs, and the MeDuSa assembly resulted in a genome length of the 4,570,268 bp based on 6 scaffolds with 95.7× coverage; the *N*_50_ value was 4,552,750 bp, and the *L*_50_ had 1 scaffold with a 57.8% G+C content in the QUAST result. CheckM showed 100% completeness and 0% contamination. PGAP annotation resulted in 4,342 genes: 4,281 coding DNA sequences (CDS), 5 rRNA genes, and 52 tRNAs genes. The TCS, ANI, and TETRA results were 0.9994, 98.65, and 0.99949, respectively, for Brucella intermedia type strain NCTC12171 (NCBI assembly accession number GCA_900454225.1). The results were 0.98984 (TCS), 82.37 (ANIb), and 0.98984 (TETRA) for Brucella daejeonensis type strain DSM 26944 (NCBI assembly accession number GCA_014199265.1). Default parameters were used for all software tools.

### Data availability.

The genome sequence has been deposited at GenBank under accession number JAIQVX000000000. Raw read data are available at SRA accession number SRR15853869, BioProject number PRJNA757421, and BioSample number SAMN20961033.
